# Using a mixture of local bone dust and morselized bone as graft materials in single- and double-level ACDF

**DOI:** 10.1186/s12891-021-04394-3

**Published:** 2021-06-02

**Authors:** Fei Ma, Shicai Xu, Yehui Liao, Qiang Tang, Chao Tang, Qing Wang, Dejun Zhong

**Affiliations:** 1 Department of Orthopedics, Affiliated Hospital of Southwest Medical University, No. 25 Taiping Street, Sichuan 646000 Luzhou, China; 2 Sichuan Provincial Laboratory of Orthopedic Engineering, No. 25 Taiping Street, Sichuan 646000 Luzhou, China

**Keywords:** Anterior cervical discectomy and fusion, Bone dust, Morselized bone, Graft materials, Surgery

## Abstract

**Background:**

Using a cage filled with local bone in anterior cervical discectomy and fusion (ACDF) can eliminate morbidities associated with autograft harvest from the iliac crest while achieving high fusion rates. However, there is still no consensus regarding the methods for using local bone grafts. This retrospective study was performed to compare the clinical and radiological outcomes of using a mixture of bone dust and morselized bone versus morselized bone alone in ACDF.

**Methods:**

A retrospective study of 228 patients affected by cervical degenerative disease who had undergone single- or double-level ACDF between January 2014 and June 2018 was performed. Nanohydroxyapatite/polyamide-66 (n-HA/PA66) combined with morselized bone was used in 111 patients (group A: single-level ACDF in 51 patients and double-level ACDF in 60 patients), whereas the n-HA/PA66 cage combined with a mixture of bone dust and morselized bone was used in 117 patients (group B: single-level ACDF in 58 patients and double-level ACDF in 59 patients). The fusion rate, extent of cage subsidence, fusion segmental height (FSH), C2-7 lordosis, segmental sagittal alignment (SSA), 10-point visual analog scale (VAS) score, and Neck Disability Index (NDI) were compared between the two groups.

**Results:**

The VAS score and NDI were significantly reduced after the operation in group A and group B. At the final follow-up, the fusion rate was 90.2 % (46/51) and 94.8 % (55/58) in patients treated with single-level ACDF in group A and group B, respectively (*p > 0.05)*. In patients treated with double-level ACDF, bone fusion was achieved in 52 patients (86.7 %) in group A and 55 patients (93.2 %) in group B (*p > 0.05)*. The fusion rate of single- and double-level ACDF was higher in patients in group B than those in group A at the 3-month, 6-month and 12-month follow-ups (*p < 0.05)*. The extent of cage subsidence after single- and double-level ACDF was lower in patients in group B (1.5 ± 0.5 mm and 2.3 ± 0.8 mm, respectively) than in those in group A (1.8 ± 0.7 mm and 2.9 ± 1.4 mm, respectively) (*p < 0.05*). There was no significant difference between the two groups in the C2-7 lordosis, FSH, SSA, VAS score, or NDI before or after the operation (*p > 0.05)*.

**Conclusions:**

Using a mixture of local bone dust and morselized bone as cage-filling materials yielded comparably good clinical outcomes as using morselized bone alone in single- and double-level ACDF. However, the mixture graft of bone dust and morselized bone was more beneficial in promoting early fusion and reducing cage subsidence.

## Introduction

Anterior cervical discectomy and fusion (ACDF), which has been practiced for over 60 years, is a classic surgical technique widely used to treat cervical myelopathy or radiculopathy [1, 2]. ACDF enables the removal of compressive anterior lesions, such as osteophytes and intervertebral discs, and the restoration of cervical spinal alignment and stability [3]. Graft materials, such as implanted cages, allografts, or autografts, should be inserted in the disc space to achieve intervertebral space support and union after removal of the disc and osteophytes [4]. Iliac bone autografts are the traditional gold standard in ACDF to achieve solid fusion. However, the graft harvesting procedure can result in a range of complications, namely, donor-site pain, hematoma, and infection [5–7].

Implanted cages, including titanium mesh cages, polyetheretherketone (PEEK) cages, and nanohydroxyapatite/polyamide66 (n-HA/PA66) cages, filled with local bone, have popularly been used as ideal and effective graft materials to avoid donor-site morbidities after iliac bone autografting during ACDF and posterior lumbar fusion [8–12]. Morselized bone obtained from removing osteophytes using a punch or curette has reportedly been used as a graft material in ACDF [8–10]. However, the bone dust (or bone shavings) obtained from removing osteophytes using a high-speed burr is often discarded without being used during ACDF.

This retrospective study was performed to explore whether using a mixture of local bone dust and morselized bone as n-HA/PA66 cage-filling materials will be more beneficial in terms of radiographic and clinical outcomes than using morselized bone alone during ACDF.

## Methods

Ethics approval was obtained before this retrospective study was initiated. Informed consent was also obtained from all subjects. In this study, all methods were performed in accordance with the relevant guidelines and regulations.

### Clinical data

Consecutive patients with degenerative cervical disease who underwent single- or double-level ACDF from January 2014 to June 2018 were included in this study. All patients underwent and were nonresponsive to conservative treatment before the operation. All operations were performed by a single surgeon (D.J.Z.). The involved segments were between C3 and C7. Patients who underwent surgery due to an infection, trauma, or tumor were excluded. Patients with a history of anterior or posterior surgical procedures, a follow-up period < 24 months, or a lack of sufficient radiographic and clinical data were also excluded. Ultimately, 228 patients were included in this retrospective study. Preoperative anteroposterior radiography, lateral radiography, flexion-extension lateral radiography, three-dimensional computed tomography (CT), and magnetic resonance imaging (MRI) of the cervical spine were performed in all patients. One hundred and eleven patients who underwent surgery between January 2014 and June 2016 were included in group A, in which local morselized bone was used as an n-HA/PA66 cage-filling material during ACDF. One hundred and seventeen patients who underwent ACDF between July 2016 and January 2018 were included in group B, in which the n-HA/PA66 cage was filled with a mixture of local bone dust and morselized bone.

### Surgical procedure and postoperative management

After general anesthesia was established, the patient was placed in the supine position. A modified right-side Smith-Robinson technique was applied in all patients [1]. After part of the disc material was removed, the vertebral body’s anterior lip was removed using a Kerrison punch, and the local morselized bone was collected. The remaining disc material and cartilage endplate were completely removed with a curette. In group A, the posterior osteophytes were removed using a Kerrison punch, high-speed burr, or/and curette. The superior and inferior bone endplates were carefully flattened using a burr. When the bone endplate seeped blood, abrading was stopped to avoid postoperative cage subsidence caused by excessive destruction of the bone endplate. After the disc height was measured, an appropriate n-HA/PA66 cage filled with local morselized bone was implanted into the disc space. An anterior cervical plate was later used to achieve immediate stabilization. In group B, the posterior osteophytes were abraded using a stainless steel burr, and the bone dust was harvested by scooping with a curette.

**Fig. 1:**
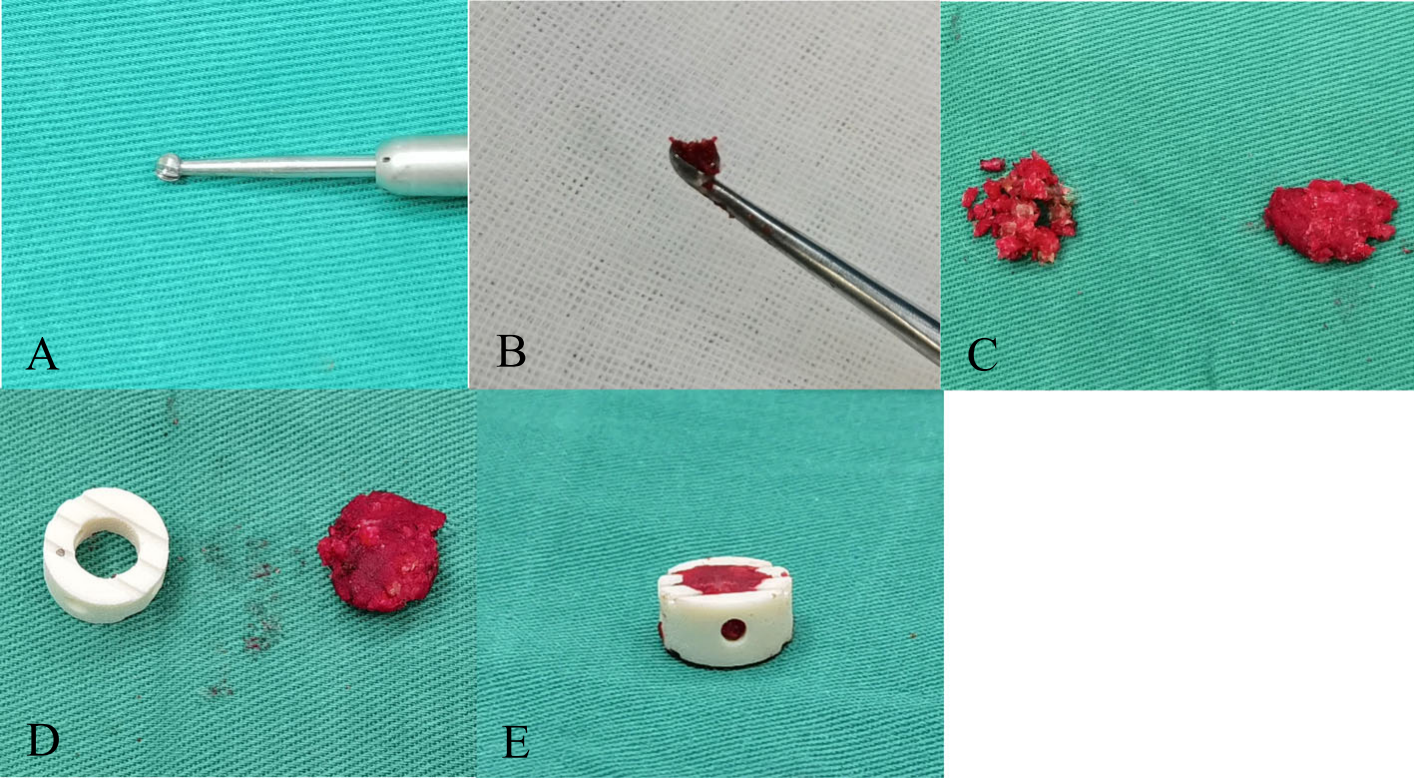
A mixture of bone dust and morselized bone was used as an n-HA/PA66 cage-filling material during the operation. **A**, **B** Posterior osteophytes were abraded using a stainless steel burr, and bone dust was harvested by scooping with a curette. **C** Morselized bone and bone dust. **D** An n-HA/PA66 cage and the bone mixture. **E** An n-HA/PA66 cage filled with the bone mixture

The difference between group A and group B was that the posterior osteophytes in group B were abraded using a high-speed burr, and the bone dust was harvested by scooping with a curette. Then, the n-HA/PA66 cage was filled with a mixture of bone dust and morselized bone in group B (Fig. 1).

A suction drain was inserted and then removed 1 or 2 days after the operation in all patients in these two groups. All patients wore a postoperative soft cervical collar for 6–12 weeks.

### Evaluation parameters and follow-up

The operative duration, blood loss, and complications were recorded and compared between the two groups. The Neck Disability Index (NDI) and the 10-point visual analog scale (VAS) score were used to assess the clinical outcomes. The Kellgren grading system [13] was used to evaluate degeneration of the surgical segments preoperatively.

A cervical spine X-ray, which was used to evaluate the fusion segmental height (FSH), segmental sagittal alignment (SSA), and C2–7 lordosis, was performed two days postoperatively, one year postoperatively, and at the final follow-up. The FSH was defined as the distance between the midpoints of the superior endplate of the cephalic vertebra and the inferior endplate of the caudal vertebra of the fused segments. The extent of subsidence was defined as the difference in the FSH between postoperatively and the final follow-up. A subsidence distance > 2 mm and > 3 mm was considered to indicate radiographic subsidence in patients treated with single-level and double-level ACDF, respectively. The SSA was defined as the angle between the line along the superior endplate of the cephalad adjacent level and the line along the inferior endplate of the caudal adjacent level. The C2–7 lordosis was defined as the sagittal Cobb angle of C2-7, and we defined lordotic angles as positive angles and kyphotic angles as negative angles. Two spinal surgeons measured all of these radiographic parameters, and the average value for each was adopted for analysis. The fusion status was evaluated by X-ray examination at three and six months after surgery. Fusion was judged by the absence of motion between the spinous processes on flexion-extension lateral radiographs, the absence of a radiolucent gap between the cage and endplate, and the presence of continuous bridging bone trabeculae at the cage-endplate junction. A three-dimensional CT scan was obtained for evaluation of the fusion status based on the five-grade criteria established by Brantigan et al. [14] at one year after surgery and the final follow-up. Grades 4 and 5 were defined as indicating bone fusion. In the evaluation and statistical analysis of fusion, patients treated with double-level ACDF were divided into three categories: no fusion (no fusion in either segment); one-level fusion (fusion in one of the segments); and two-level fusion (fusion in both segments).

### Statistical analysis

The statistical evaluation was performed using SPSS 22.0 (IBM Corp., Armonk, NY, USA). Patients treated with single-level and double-level ACDF were compared separately between the two groups. Quantitative data are presented as the mean ± standard deviation (SD). The independent t-test and the chi-square test or Fisher’s test was applied to compare the pre- and postoperative radiological and clinical data of the two groups. Paired t-tests were used for intragroup comparisons. A *p*-value of < 0.05 was considered statistically significant.

## Results

### General outcomes

There was no significant difference in age, sex, operative segment, Kellgren grade of the operative segment, medical diseases (diabetes, hypertension), smoking, operative duration, blood loss, or follow-up time between the two groups of patients treated with single- or double-level ACDF (all p > 0.05). In patients treated with single- or double-level ACDF in group A and group B, the NDI and VAS score for neck and arm pain were significantly decreased after the operation (*p* < 0.05). There was no significant difference in the NDI or VAS score between the two groups of patients treated with single- or double-level ACDF before or after the operation (*p* > 0.05). Clinical outcomes are shown in Tables 1 and 2.

**Table 1:** Patient demographic data in single-level ACDF

Variables	Group A (n = 51)	Group B (n = 58)	*P* value
**Age** (year)	48.1 ± 7.2	48.7 ± 8.5	0.73
**Male gender**	21/30	29/29	0.36
**Hypertension**	8/51	7/58	0.58
**Diabetes**	4/51	6/58	0.91
**Smoker**	11/51	9/58	0.45
**Follow-up** (month)	36.7 ± 11.3	34.8 ± 6.2	0.27
**Segements**			0.10
C3/C4	8	3	
C4/C5	9	13	
C5/C6	18	30	
C6/C7	16	12	
**Kellgren Grading Standards**			0.74
0	4	3	
1	4	8	
2	12	15	
3	23	21	
4	8	11	
**Operative time** (min)	79.5 ± 20.6	74.7 ± 18.5	0.20
**Blood loss** (ml)	62.4 ± 36.9	56.8 ± 31.5	0.40
**Complications**	1/51	1/58	1.00
**Neck VAS** (point)			
Preoperative	5.4 ± 2.2	4.9 ± 1.8	0.20
Postoperative	3.1 ± 1.9*	2.7 ± 2.0*	0.29
1 year follow-up	2.3 ± 1.4*	2.1 ± 1.3*	0.44
Final follow-up	1.5 ± 0.8*	1.6 ± 1.0*	0.57
**Arm VAS** (point)			
Preoperative	5.9 ± 2.1	6.2 ± 2.0	0.45
Postoperative	3.4 ± 1.8*	3.2 ± 1.6*	0.54
1 year follow-up	2.0 ± 1.1*	2.2 ± 1.4*	0.41
Final follow-up	1.7 ± 1.0*	1.5 ± 0.8*	0.25
**NDI**			
Preoperative	31.2 ± 13.8	29.8 ± 11.4	0.56
6 months follow-up	17.8 ± 12.4*	15.7 ± 11.2*	0.36
Final follow-up	10.3 ± 6.1*	9.7 ± 7.3*	0.65

*VAS* 10-point visual analog scale, *NDI* Neck Disability Index

* Compared with preoperative (*P* < 0.05)

**Table 2:** Patient demographic data in double-level ACDF

Variables	Group A (n = 60)	Group B (n = 59)	*P* value
**Age** (year)	51.9 ± 9.0	53.8 ± 9.9	0.27
**Male Gender**	27/33	25/34	0.92
**Hypertension**	12/60	9/59	0.50
**Diabetes**	6/60	8/59	0.55
**Smoker**	16/60	12/59	0.42
**Follow-up** (month)	38.4 ± 13.6	35.1 ± 7.9	0.11
**Segements**			0.52
C3-C5	5	2	
C4-C6	27	28	
C5-C7	28	29	
**Kellgren Grading Standards**			0.76
0	7	5	
1	11	13	
2	37	34	
3	51	46	
4	14	20	
**Operative time** (min)	110.8 ± 24.2	102.7 ± 29.5	0.10
**Blood loss** (ml)	93.8 ± 42.1	88.5 ± 37.4	0.47
**Complications**	2/60	1/59	1.00
**Neck VAS** (point)			
Preoperative	5.7 ± 2.0	5.3 ± 2.1	0.29
Postoperative	3.4 ± 1.7*	3.1 ± 1.8*	0.54
1 year follow-up	2.6 ± 1.5*	2.5 ± 1.6*	0.81
Final follow-up	1.9 ± 1.0*	2.0 ± 0.9*	0.57
**Arm VAS** (point)			
Preoperative	6.4 ± 1.9	6.7 ± 1.8	0.38
Postoperative	3.3 ± 1.6*	3.1 ± 1.5*	0.48
1 year follow-up	2.1 ± 1.2*	1.9 ± 1.1*	0.35
Final follow-up	1.8 ± 0.6*	1.7 ± 0.7*	0.40
**NDI**			
Preoperative	29.9 ± 13.1	32.4 ± 12.3	0.29
6 months follow-up	15.9 ± 10.1*	17.2 ± 11.5*	0.51
Final follow-up	11.2 ± 7.4*	10.5 ± 6.9*	0.60

*VAS* 10-point visual analog scale, *NDI* Neck Disability Index

* Compared with preoperative (*P* < 0.05)

### Radiological outcomes

#### Single-level ACDF group

**Fig. 2:**
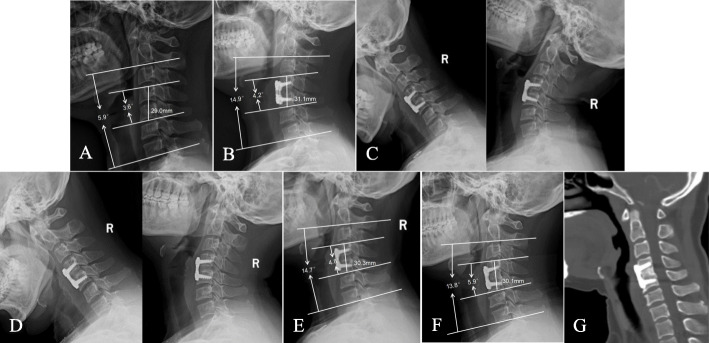
A 48-year-old female patient underwent single-level ACDF due to C4/5 disc herniation. A mixture of bone dust and morselized bone was used as an n-HA/PA66 cage-filling material during the operation. **A** Preoperative lateral radiograph of the cervical spine showing narrowing of the C4/5 disc space. **B** Postoperative lateral radiograph showing that the n-HA/PA66 cage increased the FSH, SSA, and C2–7 lordotic angle. **C**, **D** Flexion-extension lateral radiographs showing fusion at the 3-month and 6-month follow-ups. **E**, **F** Lateral radiographs showing mild cage subsidence at the 1-year and final follow-ups; the FSH, SSA, and C2–7 lordotic angle remained stable. **G** Three-dimensional CT at the 28-month follow-up time showing grade 5 fusion

There was no significant difference in the FSH, SSA, or C2-7 lordosis between the two groups preoperatively, two days postoperatively, one year postoperatively, or at the final follow-up. At the final follow-up, the subsidence distance in group A was 1.8 ± 0.7 mm, which was significantly higher than the distance of 1.5 ± 0.5 mm observed in group B (*p* = 0.011). The radiographic rate of subsidence in patients in group A was slightly higher than that of patients in group B, but there was no significant difference (*p* = 0.057). The bone fusion rate in group B was better than that in group A at 3 months (11.8 % VS. 29.3 %; *p* = 0.025), 6 months (31.4 % VS. 53.4 %; *p* = 0.020) and one year (76.5 % VS. 91.4 %; *p* = 0.032) after the operation, and without a significant difference at the final follow-up (90.2 % VS. 94.8 %; *p* = 0.577) (Fig. 2). Radiological outcomes are shown in Table 3.

**Table 3:** Radiographic outcomes in single-level ACDF

Variables	Group A (*n* = 51)	Group B (*n* = 58)	*P* value
**Fusion rate**			
3 months follow-up	6/51	16/58	0.03
6 months follow-up	16 /51	31/58	0.02
1 year follow-up	39/51	53/58	0.03
Final follow-up	46/51	55/58	0.58
**FSH** (mm)			
Preoperative	30.7 ± 3.2	29.8 ± 3.0	0.13
Postoperative	33.2 ± 3.4	32.4 ± 3.2	0.21
1 year follow-up	31.9 ± 2.9	31.2 ± 2.7	0.19
Final follow-up	31.4 ± 2.8	30.9 ± 2.7	0.35
**Subsidence** (mm)	1.8 ± 0.7	1.5 ± 0.5	0.01
**Subsidence rate**	11/51	5/58	0.06
**C2-7 lordosis** (°)			
Preoperative	10.7 ± 6.7	11.5 ± 8.1	0.57
Postoperative	14.3 ± 6.7	15.5 ± 8.6	0.39
1 year follow-up	13.9 ± 7.3	15.3 ± 8.4	0.36
Final follow-up	13.1 ± 8.1	14.8 ± 9.2	0.31
**SSA** (°)			
Preoperative	2.1 ± 4.8	2.7 ± 5.5	0.55
Postoperative	7.2 ± 3.4	8.1 ± 4.9	0.27
1 year follow-up	5.4 ± 3.1	6.7 ± 4.6	0.09
Final follow-up	5.1 ± 3.5	6.4 ± 4.0	0.08

#### Double-level ACDF group

**Fig. 3:**
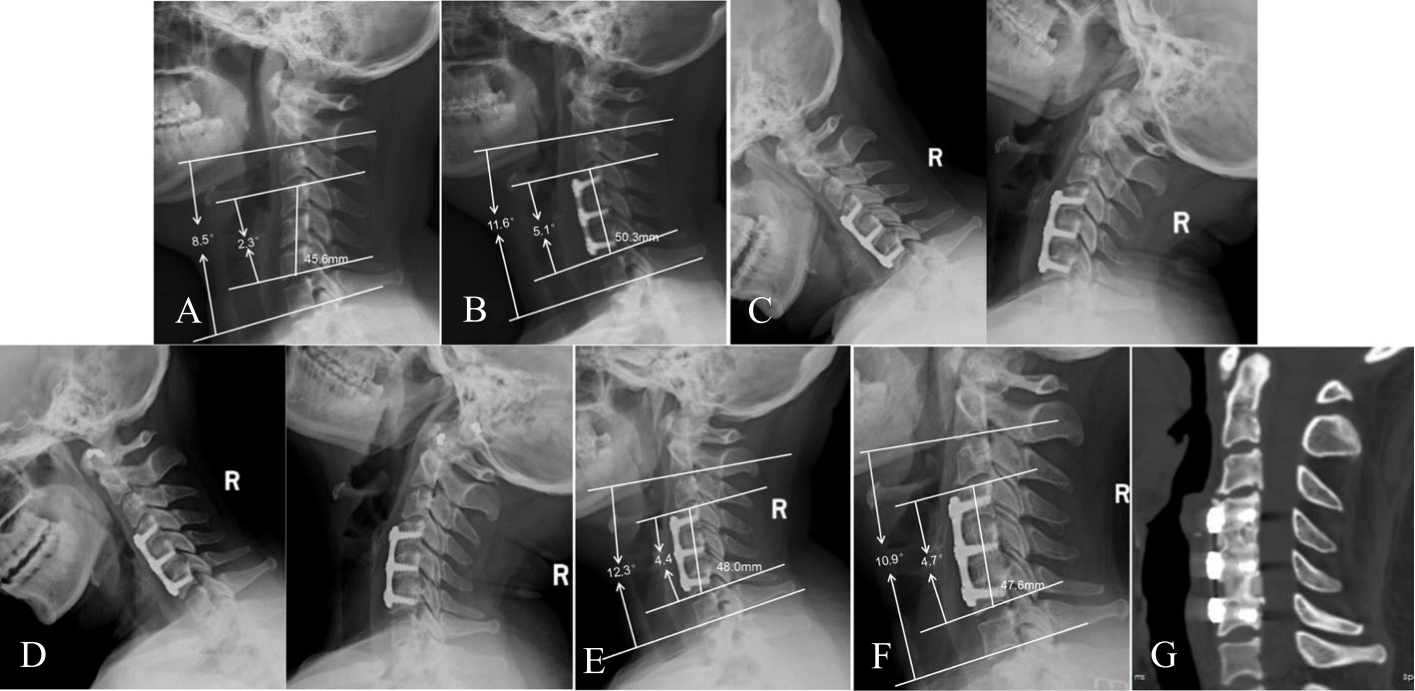
A 50-year-old female patient underwent double-level ACDF due to cervical myelopathy. A mixture of bone dust and morselized bone was used as an n-HA/PA66 cage-filling material in this patient. **A** Preoperative lateral radiograph of the cervical spine showing narrowing of the C4/5 and C5/6 disc spaces. **B** Postoperative lateral radiograph showing that the n-HA/PA66 cage increased the FSH, SSA, and C2–7 lordotic angle. **C** Three-month follow-up flexion-extension lateral radiograph showing the absence of motion between the spinous processes. **D** Six-month follow-up flexion-extension lateral radiograph showing solid fusion. **E**, **F** One-year and final follow-up lateral radiographs showing mild cage subsidence in both segments; the FSH, SSA, and C2–7 lordotic angle remained stable. **G** Three-dimensional CT at the 36-month follow-up time showing grade 5 fusion in both segments

There was no significant difference in the FSH, SSA, or C2-7 lordosis between the two groups before or after the operation (all *p* > 0.05). At the final follow-up, the subsidence distance in group A was 2.9 ± 1.4 mm, which was slightly higher than the distance of 2.3 ± 0.8 mm in group B (*p* = 0.005). There was no significant difference in the radiographic subsidence rate between the two groups (*p* = 0.138). The total bone fusion rate in group B was better than that in group A at 3 months (*p* = 0.033), 6 months (*p* = 0.014) and one year (*p* = 0.048) after the operation, and there was no significant difference at the final follow-up (*p* = 0.455) (Fig. 3). Radiological outcomes are shown in Table 4.

**Table 4:** Radiographic outcomes in double-level ACDF

Variables	Group A (*n* = 60)	Group B (*n* = 59)	*P* value
**Fusion rate**			
3 months follow-up			0.03
No fusion	53	39	
One-level fusion	4	9	
Two-level fusion	3	11	
6 months follow-up			0.01
No fusion	37	21	
One-level fusion	11	15	
Two-level fusion	12	23	
1 year follow-up			0.048
No fusion	15	6	
One-level fusion	20	17	
Two-level fusion	25	36	
Final follow-up			0.46
No fusion	3	1	
One-level fusion	5	3	
Two-level fusion	52	55	
**FSH** (mm)			
Preoperative	49.1 ± 4.5	48.9 ± 4.2	0.80
Postoperative	53.4 ± 4.9	52.7 ± 4.0	0.40
1 year follow-up	51.2 ± 4.1	51.0 ± 3.7	0.78
Final follow-up	50.5 ± 3.8	50.4 ± 3.6	0.88
**Subsidence** (mm)	2.9 ± 1.4	2.3 ± 0.8	0.01
**Subsidence rate**	17/60	10/59	0.14
**C2-7 lordosis** (°)			
Preoperative	12.9 ± 10.8	11.0 ± 12.4	0.37
Postoperative	19.1 ± 11.5	18.4 ± 11.9	0.75
1 year follow-up	17.3 ± 13.4	16.9 ± 12.7	0.87
Final follow-up	15.4 ± 10.8	17.2 ± 12.4	0.40
**SSA** (°)			
Preoperative	4.2 ± 5.4	3.9 ± 6.1	0.78
Postoperative	10.7 ± 6.1	9.3 ± 7.0	0.26
1 year follow-up	9.2 ± 5.3	8.6 ± 6.5	0.58
Final follow-up	8.5 ± 4.9	8.2 ± 5.7	0.76

*FSH* Fusion segmental height, *SSA* Segmental sagittal alignment

### Complications

One patient treated with double-level ACDF in group A developed a postoperative wound infection. A subcutaneous hematoma occurred in 1 patient treated with double-level ACDF in group B. Only one patient treated with single-level ACDF in group A underwent revision surgery because of adjacent segment degeneration 40 months after the initial ACDF procedure. All complications were resolved after the corresponding treatment. There was 1 case of screw breakage in both group A and group B, but satisfactory fusion was achieved. There were no cases of plate displacement, cage displacement, major neurological complications, or pseudarthrosis formation in either group.

## Discussion

Artificial cages filled with additional fusion materials, such as autologous iliac bone, local bone, demineralized bone matrix (DBM), bone matrix protein (BMP), or allografts, have been widely used in ACDF [6, 10, 15–17]. The use of local bone as a cage-filling material can avoid donor-site complications, including pain, hematoma, and infection, caused by using iliac bone autografts [8–10, 18]. The risk of disease transmission and the additional costs of using DBM or allografts can also be prevented [10]. Local bone is also more convenient to obtain than an iliac bone graft during surgery. Many studies have confirmed the efficacy of using local morselized bone as an artificial cage-filling material in ACDF [8–10]. A long-term follow-up study conducted by Hu et al. [8] showed that both of a PEEK cage and an n-HA/PA66 cage combined with local morselized bone in single-level ACDF yielded excellent radiographic and clinical outcomes, with a fusion rate as high as 98 %. Liu et al. [9] reported similar fusion rates for a local morselized bone graft to fill a PEEK cage compared with an autologous tricortical iliac bone graft.

In our study, patients in group A were treated with an n-HA/PA66 cage filled with morselized bone in single- or double-level ACDF. The mean NDI and VAS score were significantly improved after compared with before the operation. The mean SSA and C2–7 lordotic angle increased more than 2° after both single- and double-level ACDF. The bone fusion rate was 90.2 % at the final follow-up in patients treated with single-level ACDF and 86.7 % in patients treated with double-level ACDF. The clinical efficacy and radiographic results were satisfactory and similar to those in previous reports. However, the early solid fusion rate was relatively low, and the subsidence rate was still higher than 20 % in single- and double-level ACDF. Previously, bone dust harvested using a high-speed drill was reported to have therapeutic potential as an autologous adjunct to aid osseous fusion [19]. Histological analysis of bone dust collected during spinal surgeries has confirmed the presence of viable osteocytes in the bone dust [19, 20]. An in vitro study conducted by Eder et al. [21] revealed that bone shavings (bone dust) harvested using a high-speed burr had osteogenic potential, but this potential was lower than that of laminectomy bone chips (morselized bone) obtained using a Kerrington rongeur. In 2005, Shad et al. [22] reported the use of bone dust alone to fill the cage in ACDF. In their study, the clinical outcomes were relatively good, but the solid bone fusion rate was 77.3 % (17/22) at the one-year follow-up. This result indicates that using bone dust alone to fill the cage is not sufficient to achieve an excellent bone fusion rate at the early stage after the operation. The use of bone dust, however, provides a resource for augmenting local autografts during spinal fusion and is not associated with any significant cost or effort [20].

To further optimize the method of performing ACDF, we used a high-speed burr to abrade the posterior osteophytes and harvested the bone dust by scooping with a curette in patients in group B. During the surgery, the bone dust and morselized bone were mixed and later used to fill the n-HA/PA66 cage. In our opinion, using a mixture of bone dust and morselized bone to fill a cage is similar to filling a container with small stones and sand. In this way, the tiny space between the morselized bone can be fully filled by the bone dust, and the volume of autogenous bone in the cage can be increased. The contact area between the autogenous bone and the endplate is also more extensive than that achieved using morselized bone alone to fill the cage, facilitating the growth of blood vessels into the graft. The bone grafts are simultaneously resorbed as new bone deposition occurs within the graft site [23, 24]. Animal experiments have shown that the resorption of bone dust is faster than that of morselized bone [25]. We used a mixture of bone dust and morselized bone to fill the cage in patients in group B. This method not only controls bone absorption but also promotes bone fusion.

In the present study, patients in group B showed a fusion rate of 29.3 %, 53.4 %, and 91.4 % at 3 months, 6 months, and one year after single-level ACDF, respectively, values that are significantly higher than the corresponding rates in group A (11.8 %, 31.4 %, and 76.5 %). Similarly, patients treated with double-level ACDF in group B also had a higher fusion rate than patients in group A at 3 months, 6 months, and one year after the operation. Our results also revealed significantly greater cage subsidence after single- and double-level ACDF in group A (1.8 ± 0.7 mm and 2.9 ± 1.4 mm, respectively) than in group B (1.5 ± 0.5 mm and 2.3 ± 0.8 mm). The cage subsidence rate was reduced to 8.6% and 16.9% after single- and double-level ACDF, respectively, in group B. Although there were no significant differences in clinical outcomes or the fusion rate between the two groups at the final follow-up, it is crucial to improve the early fusion rate and reduce postoperative subsidence in ACDF. The fusion rate, fusion time, and extent of subsidence are usually used as critical indexes to evaluate the efficacy of a surgical technique [26–29]. Early fusion can also reduce the frequency of follow-up imaging examinations and increase the confidence of spinal surgeons performing this surgical technique. At present, there are still a number of studies revealing that postoperative subsidence is related to poor clinical outcomes [28–31]. Igarashi et al. [31] proposed that cage subsidence must be avoided in ACDF since it is often the cause of cervical foraminal stenosis and cage displacement.

During the operation to harvest the bone dust, saline was not used to cool the high-speed burr in our study. Park et al. [10] and Shad et al. [19] have confirmed that the use of high-speed burr without cooling in ACDF does not increase the risk of surgical complications. The related complications were also not occurred in our study. A small amount of blood seeping from the intervertebral space can reduce the temperature of the high-speed burr. Intermittent high-speed burr use during surgery can also avoid spinal cord injury caused by high temperatures from long-time driving of the high-speed burr.

The present study has several limitations. First, this study is based on a single-center, retrospective analysis, and the number of cases is small. A multicenter prospective study is required to confirm the current conclusion. Second, this study only included patients who underwent single- or double-level ACDF. Third, although both groups of patients were operated on by the same surgeon, the period of the operation was different between the two groups, which may influence the results of the study.

## Conclusions

In this study, we compared two methods of using local bone as a cage-filling material in ACDF. We found that satisfactory clinical outcomes can be obtained in patients treated with single- or double-level ACDF using a mixture of bone dust and morselized bone or morselized bone alone to fill the n-HA/PA66 cage. This result indicates that local bone grafts with an n-HA/PA66 cage is a safe alternative to iliac bone grafts in ACDF. However, an n-HA/PA66 cage filled with a mixture of bone dust and morselized bone is more beneficial for achieving earlier bone fusion and reducing subsidence. The method of harvesting bone dust is convenient and safe and does not increase the risk of surgical complications.

## Data Availability

Data will be available upon request to the first author, MF.

## Abbreviations

ACDF: Anterior cervical discectomy and fusion
PEEK: Polyetheretherketone
n-HA/PA66: Nanohydroxyapatite/polyamide66
ACF: Anterior cervical fusion
CT: Computed tomography
MRI: Magnetic resonance imaging
NDI: Neck Disability Index
VAS: Visual analog scale
FSH: Fusion segmental height
SSA: Segmental sagittal alignment
DBM: Demineralized bone matrix
BMP: Bone matrix protein
TMC: Titanium mesh cage
